# Short, Medium and Long Term Complications After Total Anatomical Shoulder Arthroplasty

**DOI:** 10.2174/1874325001711011133

**Published:** 2017-09-30

**Authors:** T.M. Gregory, B. Boukebous, J. Gregory, J. Pierrart, E. Masemjean

**Affiliations:** 1 Upper Limb and orthopaedic surgery department, Avicenne Teaching Hospital, Assistance Publique-Hôpitaux de Paris, University Paris 13, Paris, France; 2 Department of Mechanical Engineering, Imperial College, London, UK; 3 Upper Limb Surgery Unit, European Hospital Georges Pompidou, Assistance Publique-Hôpitaux de Paris, University Paris Descartes, Paris, France; 4 Department of Radiology, European Hospital Georges Pompidou, Assistance Publique-Hôpitaux de Paris, University Paris Descartes, Paris, France

**Keywords:** Arthroplasty, Shoulder, Arthroplasty, Replacement, Loosening, Glenoid placement, Radiolucent lines

## Abstract

Total shoulder arthroplasty (TSA) is an effective approach for the treatment of a variety of clinical conditions affecting the shoulder, including osteoarthritis, inflammatory arthritis and osteonecrosis, and the number of TSA implanted has grown exponentially over the past decade. This review gives an update of the major complications, mainly infections, instability and loosening, encountered after TSA, based on a corpus of recent publications and a dynamic approach: The review focuses on the causes of glenoid loosening, which account for 80% of the complication, and underlines the importance of glenoid positioning in the recovery of early shouder function and in the long term survival rate of TSA.

## 1 INTRODUCTION

Non-traumatic shoulder disorders are a common cause of morbidity. Among them, shoulder arthritis occurs in up to 7% of the population aged beyond 65 [1]. Total shoulder arthroplasty (TSA) is an effective approach for the treatment of a variety of clinical conditions affecting the shoulder, including osteoarthritis, with remarkable results on indolence and shoulder function. For the past decade, there was an exponential increase in the number of total shoulder arthroplasty. However, TSA have a relatively short survival rate as compared to knee or hip replacements, of on average 10 years [1]. The shoulder is the most mobile joint in the body. Constraints are stretching and suspension forces and not, as for the hip, compressive stresses. This is why the most common shoulder disorders are primarily diseases of the muscle-tendon unit of the shoulder and non arthritic pathologies. This is also why a dynamic musculoskeletal vision of shoulder disorders and their solutions is fundamental. The mechanical complications after total shoulder prosthesis have ​​to be analyzed with a dynamic approach.

Infections, loosening, instability, the list of complications after total shoulder prosthesis is long. This article's main objective is to update a review of the major complications encountered after total anatomic shoulder prosthesis, based on a corpus of recent publications.

## 2 METHODOLOGY

This study reviews the main complications as they may occur, chronologically.

Articles published between 1995 to 2015 and referenced in the online Pubmed database have been reviewed. The main MeSH (Medical Subject Headings) for research was « total shoulder prosthesis ». A focus was made on the intraoperative fractures, infections, primitive and iatrogenic damages to the subscapularis, loosening of the humeral and glenoid implants.

## 3 SHORT-TERM COMPLICATIONS

### 3.1 Intraoperative Fractures

**Fig. (1):**
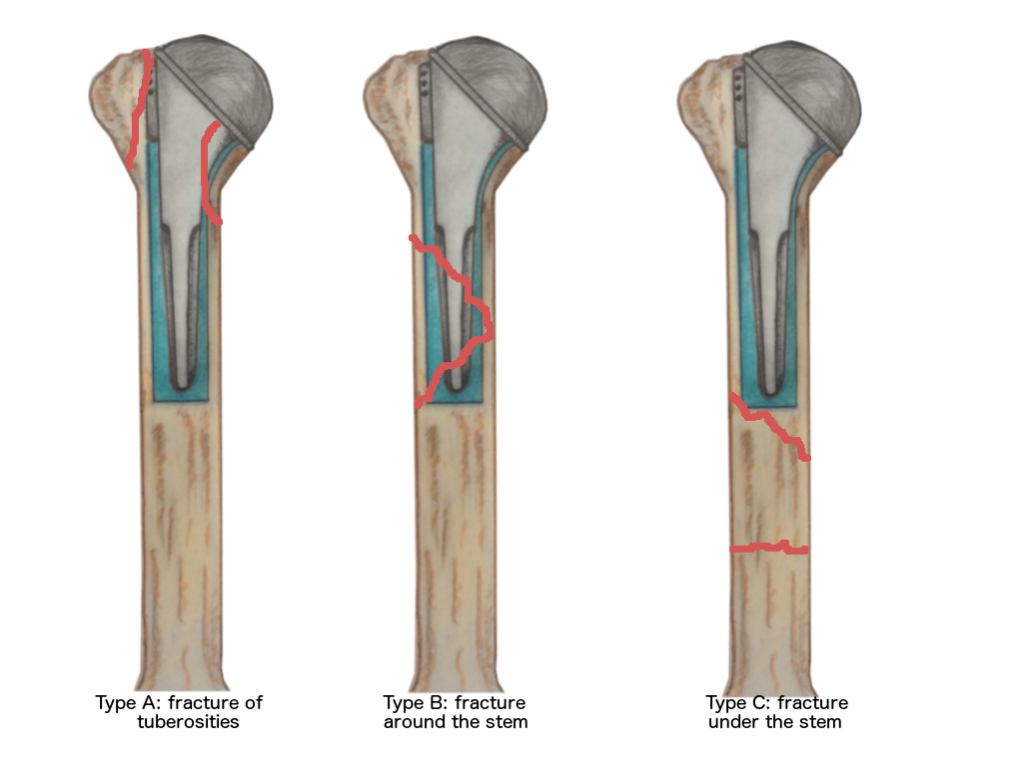
Periprosthetic humerus fracture areas according to Campbell.

The most immediate intraoperative complication is peri-prosthetic humerus fractures which account for 1.5% of the overall complications. In 2009, Athwa *et al.* [2] performed the largest series of intraoperative fractures with total shoulder arthroplasty. They analyzed 45 fractures and depicted three types of fractures: fractures of the tuberosity, the most common, especially those of the greater tuberosity, metaphyseal fractures and diaphyseal fractures. For Athwa *et al.*, these fractures healed, and, whether displaced or not, the function has been altered very little thereafter. Campbell and al [3]. described in 1998 a classification of peri prosthetic fracture, depending on their stability. Thus, fractures in regions 1 and 2 (tuberosity and metaphysis) are stable and have to receive a standard stem. The regions 3 and 4 correspond to peri and under protesthetic fractures. They are unstable and all had benefited from a long stem in the series of Campbell, with shaft approach (Fig. **1**).

Athwa *et al.* identified three fracture risk factors: female gender, press-fit stems and revisions.

### 3.2 Readmissions

Two meta-analyses were published in 2014 and target early readmissions after 30, 60 and 90 days follow up.

Mahoney and al [4]. retrospectively reviewed 376 total anatomic shoulder prostheses. With respect to total shoulder arthroplasty (TSA) 90-day readmission rate was 4,5% as compared to 8.8% for hemiarthroplasty and 6,6% for 6.6% reverse total shoulder arthroplasty. 75% of the readmissions occurred in the first 60 postoperative days. The surgical site infection was the leading cause of readmission and the second cause was secondary rotator cuff tear. All the above-mentioned complications required revision surgery. Medical complications came next.

Schairer *et al.* [5] also analysed the 30, 60 and 90 days readmission rates in a larger series of 8180 TSA.

Again, the readmission rate was significantly lower for total anatomic shoulder prosthesis (6%), that for reversed shoulder arthroplasty (11.2%) or hemi- arthroplasty (8.2%).

Most readmissions were due to medical complications (82%), and of medical causes for readmission, osteoarthritis was the most common primary diagnosis, with 11% of all readmissions. Most readmissions with osteoarthritis listed as the primary diagnosis resulted in a procedure for a separate joint, such as arthroplasty of the contralateral shoulder. Other medical diagnoses of readmission included deep venous thrombosis or pulmonary embolism (4.4% of readmissions), pneumonia (3.9%), and congestive heart failure.

Surgical complications contributed to 18% of readmissions.

Infection was the most common surgical cause for TSA readmission (5.2%), as compared to 5.0% of hemiarthroplasty and 3.2% of Reverse arthroplasty readmissions.

Readmission due to dislocation was the second most common surgical diagnosis, at 5.1% of TSA readmissions (9.3% for Reverse arthroplasty and 1.9% for hemiarthroplasty)

### 3.3 Instability, and Early Dislocations

Both above-mentioned meta-analyses reported that rotator cuff secondary tear was an early complication leading to instability. These results are consistent since it is long known that rotator cuff tears are dislocation risk factors as recently pointed out by Boileau *et al.* [6].

Warren *et al.* [7], in 2002, mentioned that TSA instability may be classified as posterior, anterior, superior, or inferior. Basic causes include malposition of the components, incorrect version of the glenoid or humeral cuts, soft tissue contractures or laxity, and cuff deficiency. These may be present as isolated or as combined deficiencies. Another cause of early anterior instability is deltoid's deficiency, which may have a neurological origin, due to plexus injury. These conditions are rare and often have a spontaneous recovery [8]. Finally, incorrect position of the glenoid component and humerus metaphysal coverage are important factors of instability.

## 4 INFECTIONS

Several infection risk factors have been identified in the literature.

Florschütz *et al.* [9], in a recent retrospective cohort of 814 patients with 350 total anatomic prosthesis, found no difference in infection rate after primary TSA and primary reverse arthroplasty in shoulders that have not undergone previous operative interventions. Infection is more likely to develop in shoulders undergoing primary reverse arthroplasty that have had one or more nonarthroplasty operative procedures. The two most common germs are Staphylococcus spp and Propionibacterium acne. In more than 90% the cases, patients developed combined infection with both germs.

Matsen III *et al.* [10] published in 2015 a series of 148 patients who underwent a total shoulder prothesis revision for glenoid loosening, despite the absence of clinical or biological infection criteria, minimum 3 years after initial intervention. Among them, 14 had deep samples, which were Propionibacterium acne positive. All were men. In 2012, while studying a cohort of 2207 patients who had total shoulder prosthesis, Cofield *et al.* [11] found that male and young patients were at higher risk to develop periprosthetic infection.

McGoldrick *et al.* [12] also suggest that the axillary region is the most likely triggering area for the development of cutaneous germs, such as propionibacterium or staphylococcus, especially among male patients. The eradication of these pathogens represents an essential potential improvement against surgical site infection. This question already arose for arthroscopy, as suggested in the recent work done by Nottage *et al.* [13].

## 5 MID-TERM COMPLICATIONS

### 5.1 Subscapularis Failure

Authors have noted that several patients have loss of internal rotation and subscapularis function on follow-up after TSA [14].

Buckley and al [15], in 2014, published a retrospective case-control cohort analyzing the sonographic appearance of sub scapularis tendon in a group of 32 patients with tenotomy and another group of 28 patients with osteotomy. The respective follow-up in both groups were 31 and 22 months. The shoulder function was assessed in each group. Four patients in the tenotomy group had pathological aspect of the sub scapular tendon as compared to none in the osteotomy group. The functional scores were similar in both groups but there was a significant decline in function in the 4 patients with pathological aspect of the subscapularis tendon.

Shi *et al.* [16], in 2015, retrospectively analyzed a case series of 5 patients who sustained failure of the letter tuberosity osteotomy after primary TSA. Two patients had a fracture without associated trauma. These LTO failures occurred between 5 and 12 weeks postoperatively. All patients required revision procedure and had chronic subscapularis altered function.

Finally, Small *et al.* [17], in 2015, retrospectively analyzed the postoperative radiographs from a cohort of 220 total shoulder prosthesis. All implants were implanted ​​with an osteotomy of the greater tuberosity. Follow up was 6 months minimum. Non-union of the osteotomy, with or without migration, was observed in 13% of the cases. Smoking and age appeared to be the predictors of non-merger. Radiography was a good diagnostic test.

Osteotomy of the lesser tuberosity or tenotomy of the subscapularis tendon are two surgical techniques commonly used for joint exposure in TSA. Both technics are associated with iatrogenic complications such tuberosity non-union or degeneration of the subscapularis, occurring in the medium term follow-up. These findings appear to be still under diagnosed. Their functional and mechanical impacts deserve to be detailed.

### 6 LONG-TERM COMPLICATIONS

Aseptic loosening of TSA implants is the main long-term complications. In a meta-analysis involving 2540 TSA, Bohsali *et al.* [8] reported that aseptic loosening glenoid constituted 39% of 14.7% complications (161 patients).

### 6.1 Loosening of The Humeral Implant

Loosening of the humeral component is a rare condition, accounting for only 7% of the complications and mainly a consequence of septic loosening [8]. However, radiolucent lines (RL) are frequently visualised around the humeral component. In 2007, Verborgt *et al.* [18] published a series of 37 TSA, with an average follow up of 9.2 years. RL around the humeral component were observed in up to 59% of the cases.

The question this study raised is whether or not RL have a detrimental impact on TSA kinematic and shoulder function. Sperling *et al.* [19] defined prognostic risk factors for symptomatic loosening. A humeral component was “at risk” when a lucent line 2 mm or greater in width was present in 3 or more of 8 zones or when at least 2 of 3 independent observers identified tilt or subsidence of the component.

Since then, several authors [18, 20] did not find significant association between these « high risk » criteria and loss of function or increase in pain. This suggests that humeral radiolucency is a natural evolutionary sign. There is little data concerning the analysis of the radiological evolution of radiolucency as a predictor of functional decline.

Finally, many studies mention humeral radiolucency for both cemented and uncemented humeral stems. Litchfield *et al.* [21], in a randomized controlled double-blind trial, reported that cemented stems performed better than non- cemented stems on the quality of life, the joint mobility and the strength with similar complication rate between the two groups.

### 6.2 Loosening of The Glenoid Implant

The glenoid loosening is the main complication after TSA, accounting for 80% of the complications [8]. As for the humerus component, RL rate around glenoid component increases over time.

### 6.3 Radiolucent Line and Osteolysis

It is generally accepted that only the progressive nature of RL is associated with loosening. However, this scalability criterion is questionable since the observation of RL varies from one observer to another and is assessed differently if only the radiological incidence differs slightly [22].

In addition, in the majority of the studies, radiolucent lines are assessed from standard radiographs in which radiolucent lines are underestimated. Yian *et al.* [23] studied a series of 47 total shoulder prosthesis. 40% of radiolucent lines visualized on CT-scan were not diagnosed on plane radiographs. More recently, Gregory *et al.* [24] demonstrated that inter-observer reliability of radiolucent lines was three times lower on standard radiographs as compared to CT-scan, and 74% of osteolysis visualized on CT-scan were not diagnosed on plane X-rays.

The CT-scan analysis of radiolucent lines is more reproducible than on standard radiographs, and it allows the analysis of peri-prosthetic osteolysis.

**Fig. (2):**
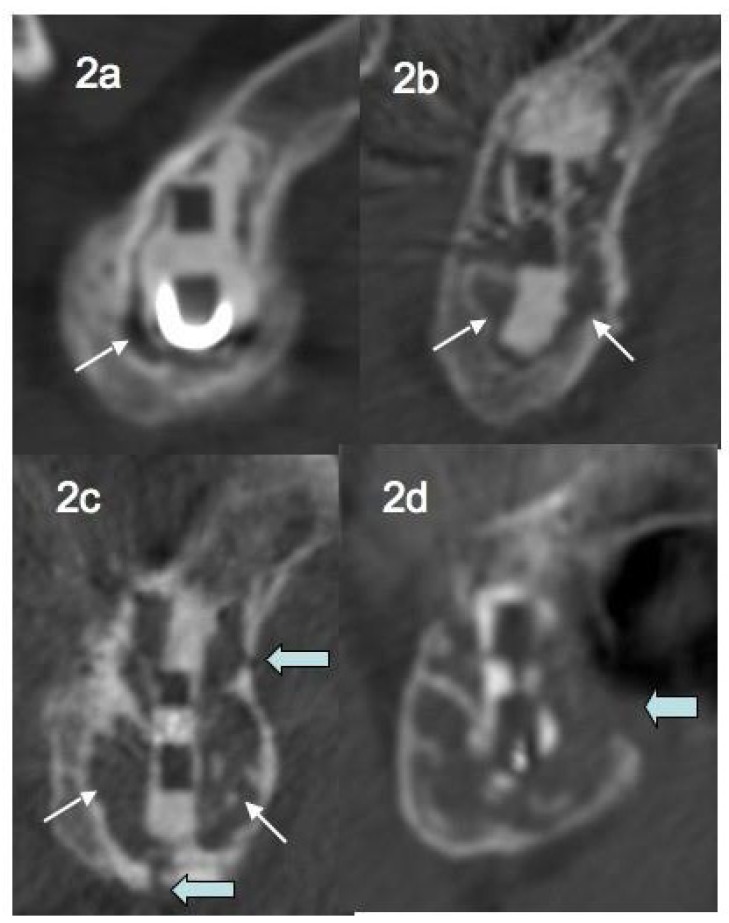
a osteolysis at an early stage, b)major osteolysis surrounding the entire fixation without reaching the cortex, c)major osteolysis associated to one or more cortical permeations, d) major osteolysis associated with lysis of the cortex.

In 2013, in a series of 68 TSA followed for a mean of 35 months, Gregory *et al.* [25] scaled peri-prosthetic glenoid osteolysis in 5 stages: absence of osteolysis, osteolysis at an early stage (located at one place of the glenoid fixation), major osteolysis surrounding the entire fixation without reaching the cortex, major osteolysis associated to one or more cortical permeations, major osteolysis associated with lysis of the cortex (Figs. **2a**, **b**, **c**, **d**).

### 6.4 Images of Loosening and Loss of Shoulder Function

These results are consistent with an alteration of the glenoid implant fixation over time. However, as for the humeral implant, these images of radiological osteolysis and RL do not always correspond to a loss of shoulder function. In 2006, in a series of total shoulder prostheses with more than 10 years follow-up, Zilber *et al.* [26] introduced the concept of “floating glenoid.”, implying a glenoid component surrounded by a large area of ​​osteolysis without major loss of function. The point of view of Gregory *et al.* [25], is that a loss of function (excluding tendon rotator cuff tear and / or traumatic loosening) is linked to a major osteolysis with cortical lysis, causing destabilization of the implant.

### 6.5 Mechanisms of Glenoid Loosening

Several factors responsible for glenoid loosening were identified in the literature.

Wirth *et al.* [27] performed an histological analysis of the membrane around three total shoulder prosthesis revised for aseptic loosening and accompanied by radiological evidence of osteolysis. They found, in each case, an identical process of polyethylene granuloma, which is responsible for aseptic loosening of hip replacements Fig. (**3**). Moreover, measurement of the wear rate of the polyethylene was carried out in-vivo by CT-scan method. In a study involving « Neer 2 » total prosthesis, Emery *et al.* [28] evaluated the polyethylene wear to 0.38 mm per year, for a total 4mm implant thickness.

Nho *et al.* [29] in 2009, analysed a retrospective series of 78 revised glenoid implants. Thirty-five had glenoid deformations compatible with the application of eccentric constraints: peripherical deformation, occurrence of an eccentric wear cavity or delaminated appearance of the polyethylene Fig. (**3**). A peripheral abrasion was observed in 29 glenoids, reflecting an impingement between the edge of the glenoid rim and the humeral metaphysis. Seven implants were fractured. Thus, polyethylene wear debris leading to aseptic loosening of glenoid implant is two folds: wear due to eccentric stresses apply on to the glenoid by the humeral head, also called “rocking horse” effect, and peripheral impingement of the glenoid.

Loosening mechanism, so-called « rocking horse », was described by Franklin and al in 1988 [30]. It consists firstly in eccentric stresses applied to the glenoid, increasing asymmetric wear of the polyethylene. On the other hand in the cement-bone interface of the prosthesis, compressive stresses and shear on one side and distraction stress on the other side, promote the mechanical loosening of the implant in the cement-bone interface.

For Franklin et al, failure mechanism of glenoid implant was associated with rotator cuff tear, and the humeral head applies eccentrical forces onto the glenoid rim. However, for Augereau *et al.* [31], partial or isolated supraspinatus tear does not contraindicated the use of TSA.

### 6.6 Importance of Glenoid Positioning

Positioning of glenoid implant also proved to be critical for TSA results. In 2012, Gregory *et al.* published a series of 29 total shoulder prostheses [32], for which the glenoid orientation was assessed on preoperative and postoperative CT-scans. On post-operative CT-scan, the error of glenoid placement related to the standard position was on average 12 degrees in each space direction (version, inclination or rotation, Fig. (**4**)). With respect retroversion, the glenoid was “properly” positioned, *i.e.* positioned in the maximum bone axis of the glenoid vault, in only 25% of the cases. Several authors have studied the effect of positioning errors on the implants loosening. Farron *et al.* [33], in 2006, showed that retroversion of the glenoid implant involved a posterior subluxation of the humeral head. Then Ho *et al.* [34], in 2013, found an association between implant retroversion and appearance of osteolysis around the fixation. The posterior instability of the shoulder has a multi factorial origin. One of the causes is a glenoid implant malposition [3].

Finally, quality of primary fixation of the glenoid implant appears to be a major factor involved in loosening. In 2009, Gregory *et al.* performed a cadaveric study involving six models of glenoid implants [35], subjected to artificial constraints. They demonstrated that failure appears first at the cement-implant interface. This mechanical failure is rarely associated with loosening because of the retentive nature of cement. Still in the same study, a second failure in the cement-bone interface secondarily appeared and progressed more or less quickly toward central fixation, leading to loosening. The rate of appearance and spread of this depends on the quality of the cementing technic. For example, poor washing and drying of the bone before setting up cement, or without cementing pressure, are both low quality items. Another example is that loosening appears earlier in the rheumatoid arthritis than simple osteoarthritis. One reason, supported by Strauss *et al.* [36], is the existence of an inflammatory bone of lower quality.

## CONCLUSION

Analysis of complications after total shoulder arthroplasty cannot be done without a dynamic approach. Whether short, medium or long term, an imbalance of stresses onto the glenoid implant is responsible for premature wear and instability. Subscapularis early failure is correlated with early instability.

The major complication after TSA is aseptic glenoid loosening. Radiolucent line and osteolysis are better appreciated on CT-scan than on plane X-rays. However, radiological signs of loosening are not always associated with loss of function, especially when the vault cortex is intact and if the glenoid is implanted without error. The loosening mechanisms are both « rocking horse » effect and impingement between the edge of the glenoid rim and humeral metaphysis. The positioning of the glenoid implant is a major challenge. Development of technical operation supports for the positioning of glenoid implants is critical to improve TSA results.

## Figures and Tables

**Fig. (3):**
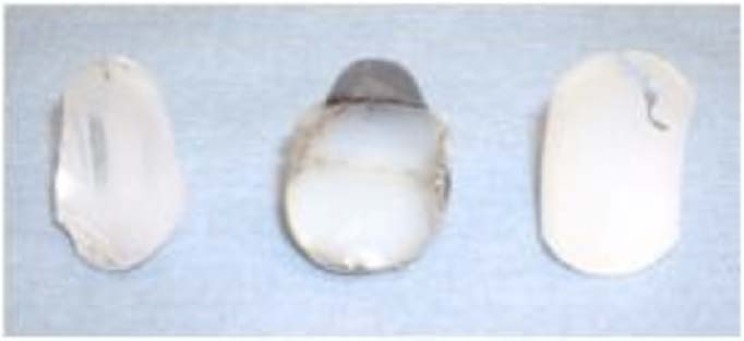
periphery deformation, appearance of an eccentric wear cavity or delaminated appearance of the polyethylene.

**Fig. (4):**
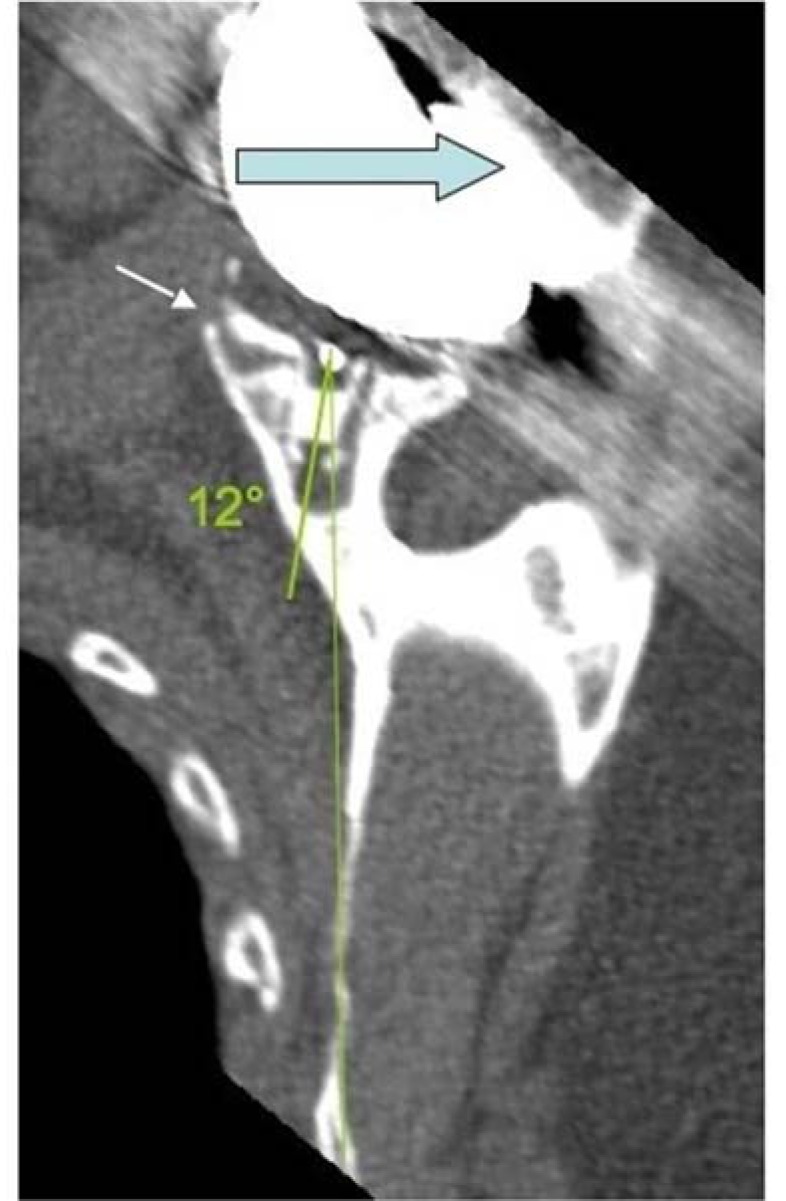
Positioning in retroversion (12°) of the glenoid leading to rocking horse effect in the AP direction with humeral head posterior subluxation and ultimately to glenoid loosening.
